# Use of Doubly Labeled Water to Validate a Physical Activity Questionnaire Developed for the Japanese Population

**DOI:** 10.2188/jea.JE20100079

**Published:** 2011-03-05

**Authors:** Kazuko Ishikawa-Takata, Yoshihiko Naito, Shigeho Tanaka, Naoyuki Ebine, Izumi Tabata

**Affiliations:** 1 Program of Health Promotion and Exercise, National Institute of Health and Nutrition, Tokyo, Japan; 2 School of Human Environmental Sciences, Mukogawa Women’s University, Hyogo, Japan; 3 Faculty of Health and Sports Science, Doshisha University, Hyogo, Japan; 4 Faculty of Sport and Health Science, Ritsumeikan University, Shiga, Japan

**Keywords:** physical activity questionnaire, doubly labeled water, physical activity, energy expenditure

## Abstract

**Background:**

No study has attempted to use the doubly labeled water (DLW) method to validate a physical activity questionnaire administered to a Japanese population. The development and refinement of such questionnaires require that physical activity components related to physical activity level be examined.

**Methods:**

Among 226 Japanese men and women 20 to 83 years of age, total energy expenditure (TEE) was assessed using the Japan Arteriosclerosis Longitudinal Study Physical Activity Questionnaire (JALSPAQ), and the results were compared with TEE measured by the DLW method as a gold standard. Resting metabolic rate (RMR) was measured using the Douglas Bag method.

**Results:**

The median TEE by DLW and physical activity level (PAL: TEE/RMR) were 11.21 MJ/day and 1.88, respectively, for men, and 8.42 MJ/day and 1.83 for women. JALSPAQ slightly underestimated TEE: the differences in mean and standard error were −1.15 ± 1.92 MJ/day. JALSPAQ and DLW TEE values were moderately correlated (Spearman correlation = 0.742, *P* < 0.001; intraclass correlation coefficient = 0.648, *P* < 0.001), and the 95% limit of agreement was −4.99 to 2.69 MJ. Underestimation of TEE by JALSPAQ was greater in active subjects than in less active subjects. Moderate and vigorous physical activity and physical activity during work (ie, occupational tasks and housework) were strongly related to physical activity level. However, the physical activity components that differentiated sedentary from moderately active subjects were not clear.

**Conclusions:**

Physical activity level values on JALSPAQ and DLW were weakly correlated. In addition, estimation of TEE in active subjects should be improved, and the use of a questionnaire to differentiate activity in sedentary and moderately active subjects must be reassessed.

## INTRODUCTION

Accurate assessment of physical activity level is fundamental in epidemiological studies that examine the effect of physical activity on disease prevention and health promotion. Although there are several methods for estimating physical activity level, questionnaires are the most common assessment tool in such studies. Many types of physical activity questionnaires are used in epidemiological studies, but a validation study of such questionnaires suggested that the reliability and validity of measurements of habitual physical activity are quite low.^1^^–^^3^ In addition, Neilson et al suggested that the ability of physical activity questionnaires to predict total energy expenditure (TEE) is limited. Westerterp et al suggested that questionnaires are satisfactory as an instrument for ranking physical activity level, but not as tools for assessing absolute TEE.^4^ We previously examined the International Physical Activity Questionnaire (IPAQ) and reported that it was difficult to distinguish sedentary from moderately active individuals in the Japanese population.^5^ Although the IPAQ was developed for international use, we maintain that questionnaires designed to suit each country or culture would increase the validity of assessments of physical activity level. The Japan Arteriosclerosis Longitudinal Study Physical Activity Questionnaire (JALSPAQ) was developed to assess physical activity in the Japan Arteriosclerosis Longitudinal Study.^6^^,^^7^ This questionnaire was developed using data from physical activity records for the Japanese population and included detailed questions on occupational work, housework, and leisure-time physical activity.

The doubly labeled water (DLW) method is an excellent method for measuring TEE in free-living subjects over a period of 1 to 2 weeks^8^ and is often used as a gold standard to validate field methods of assessing physical activity levels. However, to our knowledge, only our previous study^5^ has used it to examine the validity of a questionnaire used for the Japanese population.

The primary objective of this study was to use the DLW method as the gold standard to validate a physical activity questionnaire developed for the Japanese population. To aid in the development of a valid physical activity questionnaire for Japanese, the secondary objective was to identify the physical activity component that had the greatest impact on physical activity level (PAL).

## METHODS

### Subjects

The study participants were 226 Japanese men and women age 20 to 83 years (mean ± standard deviation, 50.4 ± 17.1 years) who volunteered at community health care centers and workplaces or enrolled via the internet homepage of our institute. The inclusion criteria of the present study were as follows: absence of any condition affecting energy or water metabolism (eg, thyroid or kidney disease), not pregnant or breast-feeding, residence in home prefecture 2 weeks before and during the study, not on weight-loss or treatment diet, and not consuming more than 40 grams of alcohol per day. The occupations of the participants were homemaker (*n* = 59), office worker (*n* = 57), shipbuilder (*n* = 17), shop assistant (*n* = 14), no regular work (*n* = 14), nurse (*n* = 13), teacher (*n* = 11), salesperson (*n* = 11), factory worker (*n* = 6), clinical examination technician (*n* = 5), physiotherapist (*n* = 4), and other (*n* = 12, cleaner, gardener, dietitian, priest, sports instructor, carpenter, etc.). We were unable to randomly select subjects according to physical activity level. Over the entire assessment period, the participants were carefully instructed to maintain their normal daily activities and eating patterns and to make no conscious effort to lose or gain weight.

### Study protocol

This study was approved by the Ethics Committee of the National Institute of Health and Nutrition in Japan. All subjects gave their informed consent in writing before the investigation was begun. TEE was estimated over 1 or 2 weeks, depending on the 2 half-lives of the isotopes used in the DLW method. Body mass and height were measured in the fasting state before administering the dose of DLW and on the last day of the study. On the first day of the study period, baseline urine was collected, and measurements of resting metabolic rate (RMR) and DLW dosing were obtained. The physical activity questionnaire and dietary assessment were completed between the 10th and 12th day of the study period and were checked by the researchers on the last day.

### Measurement of resting metabolic rate

Subjects were instructed to refrain from moderate to vigorous physical activity for 24 hours, to fast at least 12 hours, and to get sufficient sleep before the measurements. They were instructed to arrive at the laboratory between 8AM and 9AM. After arrival, they rested quietly in the supine position for 30 minutes before the measurements. Using a mask connected to a Douglas bag, expired gas was collected twice for 10 minutes, with a 1-minute interval between collections. During all RMR measurements, the room temperature was maintained at approximately 24°C. Subjects were lying down and fully awake during the measurements. They were also free from emotional stress and were familiar with the apparatus used. The volume of expired air was measured with a certified gas meter (DC-5, Shinagawa, Tokyo, Japan), the accuracy and precision of which were maintained within 1% of the coefficient of variation (CV). Concentrations of oxygen and carbon dioxide were measured with a mass spectrometer (ARCO-1000, Arco Systems, Chiba, Japan). The precision of expired gas measurement was 0.02% for oxygen and 0.06% for carbon dioxide. RMR was calculated using Weir’s equation.^9^

### DLW energy measurement

After providing a baseline urine sample, a single dose of approximately 0.06 g/kg body weight of ^2^H_2_O (99.8 atom%, Cambridge Isotope Laboratories, MA, USA) and 1.4 g/kg body weight of H_2_^18^O (10.0 atom%, Taiyo Nippon Sanso, Tokyo, Japan) was given orally to each subject. Then subjects were asked to collect urine samples at 8 predetermined times during the study period, at the same time of day. Except for the baseline collection, all urine samples were collected by the participant, and the time of sampling was recorded. All samples were stored by freezing at −30°C in airtight parafilm-wrapped containers and then analyzed in our laboratory.

Gas samples for the isotope ratio mass spectrometer (IRMS) were prepared by equilibration of the urine sample with a gas. CO_2_ was used to equilibrate ^18^O, and H_2_ was used for ^2^H. Pt catalyst was used for equilibration of ^2^H. The gas sample of the CO_2_ and H_2_ was analyzed by IRMS (DELTA Plus; Thermo Electron Corporation, Bremen, Germany). Each sample and the corresponding reference were analyzed in duplicate. The average standard deviations for the analyses were 0.5‰ for ^2^H and 0.03‰ for ^18^O. TEE was expressed as mean TEE per day over the study period.

### Calculations of isotopic abundance and TEE

The ^2^H and ^18^O zero-time intercepts and elimination rates (k_H_ and k_O_) were calculated using a least-squares linear regression on the natural logarithm of isotope concentration as a function of the elapsed time from dose administration. Zero-time intercepts were used to determine the isotope pool sizes. Total body water (TBW) was calculated from the mean value of the isotope pool size of ^2^H divided by 1.041 and that of ^18^O divided by 1.007. The mean ko/kd of the present study was 1.28 ± 0.06 (range, 1.15–1.56). All ko/kd values were maintained within the recommended range (1.1 to 1.7) for quality control of the analysis, as recommended by the International Atomic Energy Agency.^10^ rCO_2_ was calculated as follows: rCO_2_ = 0.4554 × TBW × (1.007ko − 1.041k_H_). Calculation of TEE (kcal/day) was performed using a modified Weir’s formula based on the CO_2_ production rate (rCO_2_) and food quotient (FQ).^9^ FQ was calculated from the dietary survey during the study period. The calculation assumed that under conditions of perfect nutrient balance, the FQ must equal the respiratory quotient (RQ).^11^^–^^13^ The average FQ of each occupational group was used for each group (FQ = 0.85–0.95). However, FQ values stratified by occupational group, sex, and age were not significantly different. Physical activity level (PAL) was calculated as TEE/RMR.

### Physical activity questionnaire

The physical activity questionnaire developed for the Japan Arteriosclerosis Longitudinal Study (JALSPAQ) was used in this study.^6^^,^^7^ This questionnaire comprises 14 questions on occupation, locomotion, housework, sleep time, and leisure-time physical activities. In this questionnaire, occupational work was assessed as duration of sitting, standing, walking, and heavy work. Heavy work was defined as lifting more than 10 kg or manual labor of similar intensity. Leisure-time physical activity was assessed by type, duration, and frequency. Questionnaire data were converted to the intensity of each physical activity expressed in metabolic equivalents (METs), according to the Compendium by Ainsworth et al, and summarized as METs·h/day and energy expenditure.^14^ In the present study, we used TEE per day, METs·h/day, and PAL as indices of physical activity level from JALSPAQ. Duration of light (<3 METs), moderate (3–5.9 METs), and vigorous (≥6 METs) physical activities was calculated for all physical activities (including occupational activity, housework, and leisure-time physical activity), as well as for leisure-time physical activity only. Working time, including occupational and housework time, was divided into the duration of sitting (<2 METs), standing (2 to <3 METs), walking (3 to <6 METs), and heavy work (≥6 METs), including housework. We calculated the durations of occupational activity and housework together because their frequencies and durations were quite complicated.

### Dietary assessment

Dietary habits were assessed by using a brief self-administered diet history questionnaire (BDHQ)—a 4-page structured questionnaire that requested information on the consumption frequencies for a total of 56 food and beverage items, with specified serving sizes described in terms of the servings commonly consumed in the general Japanese population.^15^ Energy and macronutrient intakes were calculated using a computer algorithm for the BDHQ, which was based on the Standard Tables of Food Composition in Japan. FQ was calculated by using the equation of Black et al.^11^

### Statistical analysis

Statistical analyses were performed using SPSS for Windows (version 16.0J; SPSS Inc., IL, USA). Physical characteristics are classified using the sex and age groups outlined in the Dietary Reference Intake (DRI) of Japan. The estimated energy expenditure data were generally not normally distributed; therefore, medians and interquartile ranges are used to describe these results. Sex and age-group differences were compared using 2-way analysis of covariance. The Bonferroni procedure was used as the post-hoc test. The relation between TEE as estimated by DLW and JALSPAQ was expressed as Spearman correlations, intraclass correlation coefficient (ICC), and 95% limits of agreement (95% LOA: mean difference ± 2 × SD of the mean difference). Bland-Altman plots were also created to evaluate the differences between the 2 methods. To examine the type of physical activities that affected physical activity level, we used 1-way analysis of covariance, Pearson’s correlation coefficients, and partial correlation coefficients adjusted for sex and age group.

## RESULTS

The physical characteristics of the subjects are shown in Table 1. Body weight did not change significantly during the study period (*P* = 0.313). Among all subjects, 2.8% of men and 6.8% of women were classified as lean (body mass index [BMI] <18.5 kg/m^2^), and 31.5% of men and 17.8% of women were classified as obese (BMI >25 kg/m^2^) according to the criteria for Japanese.^16^ The average TBW was 37.3 ± 7.1 kg in men and 25.9 ± 2.8 kg in women. When 73.2% was defined as the proportion of water in fat-free mass, the percent of fat mass was 24.3 ± 6.1% in men and 33.4 ± 7.0% in women.^17^ Three men aged 30 to 49 years had a body weight higher than 100 kg; however, they were fit and their percent of fat mass was less than 25%. In addition, in the assessment of TEE by DLW and JALSPAQ, they did not significantly differ from other subjects.

**Table 1.** Characteristics of study subjects

Age group, years	*n*	Age (years)	Height (cm)	Body weight			BMI (kg/m^2^)	TBW (kg)

				pre (kg)	post (kg)	change (kg)		
Male								
20–29	18	25.0 ± 2.5	171.5 ± 6.0	62.1 ± 7.9	62.3 ± 8.0	0.2 ± 0.7	21.1 ± 2.0	36.4 ± 3.7
30–49	42	36.7 ± 5.3	173.8 ± 6.6	74.8 ± 16.7	74.9 ± 16.6	0.0 ± 1.1	24.6 ± 4.7	41.8 ± 8.3
50–69	31	60.2 ± 6.5	163.8 ± 6.6	63.9 ± 8.1	64.0 ± 8.3	0.1 ± 0.9	23.8 ± 2.4	34.5 ± 4.1
≥70	17	75.1 ± 4.0	162.1 ± 5.0	60.7 ± 8.1	60.8 ± 8.2	0.2 ± 0.9	23.1 ± 2.7	32.0 ± 4.2
Female								
20–29	8	25.3 ± 2.4	157.0 ± 3.9	51.3 ± 2.5	51.2 ± 2.5	−0.1 ± 0.8	20.9 ± 1.6	25.5 ± 1.5
30–49	42	38.7 ± 4.4	158.0 ± 5.4	53.7 ± 8.3	53.7 ± 8.3	0.0 ± 0.7	21.5 ± 3.2	26.9 ± 3.1
50–69	49	62.0 ± 5.1	154.0 ± 4.6	54.6 ± 7.8	54.7 ± 7.9	0.1 ± 0.7	23.0 ± 3.2	25.8 ± 2.7
≥70	19	73.4 ± 3.9	148.0 ± 4.4	50.2 ± 6.1	50.1 ± 6.1	0.1 ± 0.6	22.9 ± 2.8	24.1 ± 2.0

All values are mean ± SD, unless otherwise indicated.

BMI: body mass index; TBW: total body water measured by doubly labeled water method.

The medians plus interquartiles for RMR, TEE, and PAL by DLW, TEE by questionnaire, and the differences between the 2 methods are shown by sex and age group in Table 2. The respective medians of TEE and PAL were 11.21 MJ/day and 1.88 for men and 8.42 MJ/day and 1.83 for women. PAL significantly differed by age group, but not by sex. PAL in subjects older than 70 years was significantly higher than in those aged 30 to 49 years (*P* = 0.016) and 50 to 69 years (*P* < 0.001). JALSPAQ slightly underestimated TEE, with differences in mean and standard error of the mean of −1.15 ± 1.92 MJ/day and −0.020 ± 0.030 MJ/kg/day. TEE values by JALSPAQ and DLW were moderately correlated (Spearman correlation = 0.742, *P* < 0.001; ICC = 0.648, *P* < 0.001). The 95% LOA was −4.99 to 2.69 MJ. The absolute difference between TEE values by DLW and JALSPAQ was significantly greater in men than in women, but the percent difference was not significantly different. The Spearman correlation coefficient and ICC for PAL were 0.423 (*P* < 0.001) and 0.332 (*P* < 0.001), respectively, and the 95% LOA for PAL was −0.86 to 0.46. Use of Bland-Altman plots to compare TEE and PAL by DLW and JALSPAQ suggested that TEE tended to be underestimated in subjects with higher TEE (Spearman correlation, −0.201; *P* = 0.002); however, most values were within the 2 SD of the difference in TEE as determined by the 2 methods (Figure). PAL was not underestimated even in subjects with higher PALs (Spearman correlation, −0.011; *P* = 0.866); however, individual differences were widely distributed.

**Figure.**
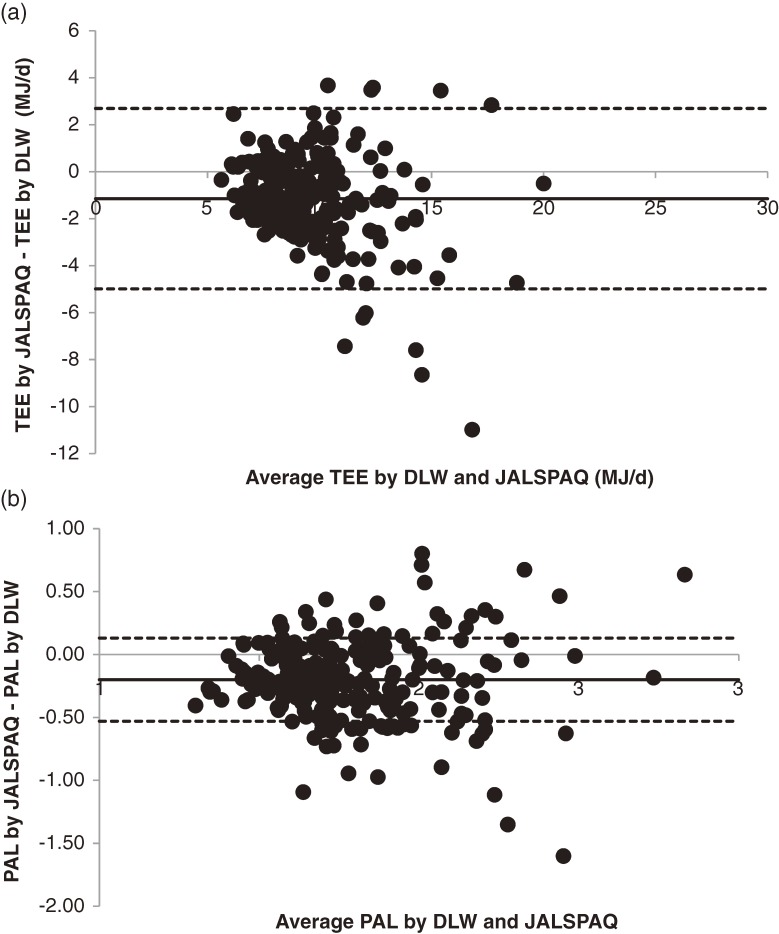
Bland-Altman plots of total energy expenditure (TEE) and physical activity level (PAL). (a) Comparison of mean TEE estimated by the doubly labeled water (DLW) method and the Japan Arteriosclerosis Longitudinal Study Physical Activity Questionnaire (JALSPAQ), and the difference in TEE as estimated by the 2 methods. (b) Comparison of mean PAL by DLW and JALSPAQ, and the difference in PAL as estimated by the 2 methods. Solid lines indicate the mean difference, and the broken lines indicate 2 SD limits.

**Table 2.** Resting metabolic rate (RMR) and total energy expenditure (TEE) measured by doubly labeled water (DLW) method and questionnaire

Age group, years		RMR (MJ/day)	TEE by DLW (MJ/day)	PAL	TEE by JALSPAQ (MJ/day)	Difference between DLW and JALSPAQ	

						(MJ/day)	(%)
Male							
	20–29	6.27 (0.92)	12.00 (0.19)	1.89 (0.35)	9.60 (2.12)	−1.69 (2.89)	−15.7 (23.0)
	30–49	6.72 (1.53)	12.88 (4.64)	1.87 (0.45)	11.14 (2.85)	−1.18 (3.30)	−9.5 (20.3)
	50–69	5.50 (1.30)	10.81 (2.11)	2.08 (0.55)	9.18 (1.61)	−2.02 (1.99)	−18.1 (17.5)
	≥70	5.76 (1.41)	11.76 (3.59)	2.11 (0.52)	8.03 (1.65)	−0.97 (2.34)	−12.2 (21.0)
Female							
	20–29	4.73 (0.27)	8.10 (1.18)	1.86 (0.22)	7.43 (1.01)	−1.09 (1.85)	−13.2 (22.3)
	30–49	4.83 (0.82)	8.82 (1.80)	1.84 (0.32)	7.33 (1.75)	−1.26 (1.73)	−14.9 (19.1)
	50–69	4.58 (0.95)	8.53 (1.42)	1.86 (0.37)	8.12 (1.28)	−0.43 (1.76)	−5.3 (20.4)
	≥70	4.62 (0.99)	8.56 (0.86)	1.86 (0.41)	7.08 (1.33)	−0.36 (1.68)	−5.2 (23.3)

*P* value	Sex	<0.001	<0.001	0.067	<0.001	0.003	0.071
	Age group	<0.001	<0.001	<0.001	<0.001	0.335	0.370
	Sex by age	0.010	0.004	0.481	<0.001	0.591	0.188

All values are median (interquartile), unless otherwise indicated.

PAL: physical activity level (TEE/RMR); JALSPAQ: Japan Arteriosclerosis Longitudinal Study Physical Activity Questionnaire.

Using PAL determined using TEE measured by DLW, the subjects were divided into 3 groups according to Dietary Reference Intake (Table 3).^18^ The proportions of active (PAL >1.9), moderately active (PAL 1.6 to <1.9), and sedentary (PAL <1.6) individuals were 45.4%, 43.5%, and 11.1% in men, respectively, and 40.7%, 41.5%, and 17.8% in women. TEE by JALSPAQ in the sedentary group was significantly lower than in moderately active and active adults. Total METs assessed by JALSPAQ was lower in sedentary and moderately active individuals than in active individuals. The differences between the 2 methods in the TEE of sedentary and moderately active adults were significantly smaller than in active adults. The total duration of each intensity of physical activity, including occupational and housework activity and leisure-time physical activity, was compared among physical activity levels. The duration of moderate and vigorous physical activity in sedentary and moderately active adults was significantly shorter than in active adults. When we compared only leisure-time physical activity, there was no difference in duration of physical activity. Regarding physical activity during work, duration of walking was significantly shorter in sedentary individuals than in moderately active and active individuals. In addition, walking duration was significantly shorter in moderately active adults than in active adults. The proportion of heavy work differed significantly among groups; greater activity was associated with heavier work.

**Table 3.** Total energy expenditure (TEE) and duration of each activity among groups by physical activity level

	Physical activity level			*P*

	I Sedentary	II Moderately active	III Active	
TEE by DLW (MJ/day)	8.11 (1.39)^a,b^	9.18 (2.29)^b^	10.76 (4.25)	*<0.001*
TEE by questionnaire (MJ/day)	7.78 (1.21)^b,c^	8.45 (2.87)	8.90 (3.06)	0.006
Total METs (METs·h/day)	33.5 (4.1)^b^	34.4 (4.8)^b^	35.8 (6.4)	<0.001
Difference in TEE between DLW and PAQ (MJ/day)	−0.07 (0.50)^b^	−0.80 (1.62)^b^	−2.02 (2.23)	<0.001
Difference in TEE between DLW and PAQ (%)	−0.9 (15.3)^b^	−8.4 (17.6)^b^	−19.1 (19.0)	<0.001
Total duration of physical activity (h/day)				
Light (<3 METs)	3.41 (3.58)	4.14 (3.50)	4.16 (3.72)	0.155
Moderate (3–5.9 METs)	1.65 (1.81)^b^	2.06 (2.07)^b^	2.53 (3.89)	<0.001
Vigorous (≥6 METs)	0.00 (0.09)^b^	0.00 (0.20)^a^	0.0 (0.54)	0.007
Duration of leisure-time physical activity (h/day)				
Light (<3 METs)	0.00 (0.26)	0.00 (0.07)	0.00 (0.09)	0.766
Moderate (3–5.9 METs)	0.01 (0.17)	0.02 (0.23)	0.03 (0.27)	0.965
Vigorous (≥6 METs)	0.00 (0.08)	0.00 (0.02)	0.00 (0.00)	0.556
Duration of work (h/day)				
Sitting	0.00 (2.86)	1.55 (4.61)	0.00 (4.29)	0.129
Standing	1.75 (2.20)	1.42 (2.14)	2.00 (2.85)	0.176
Walking	0.25 (0.86)^b,c^	0.54 (1.90)^b^	1.00 (3.07)	<0.001
Proportion of subjects participating in heavy work (%)	6.1	24	36.1	0.003

TEE: total energy expenditure; DLW: doubly labeled water; MET: metabolic equivalent; PAQ: physical activity questionnaire.

All values are median (interquartile), unless otherwise indicated.

^a^*P* < 0.05 as compared with physical activity level III.

^b^*P* < 0.01 as compared with physical activity level III.

^c^*P* < 0.01 as compared with physical activity level II.

Regarding the types of physical activity that were correlated with PAL, correlation coefficients and partial correlation coefficients adjusted for sex and age group are shown in Table 4. Duration of total, moderate, and vigorous physical activity were weakly correlated with PAL. However, duration of leisure-time physical activity was not correlated with PAL. During working time, duration of standing, walking, and heavy work were weakly correlated with PAL.

**Table 4.** Correlation coefficients for physical activity level (as measured by doubly labeled water method) and duration of physical activities

	Correlation coefficient	*P* value	Partial correlation coefficient	*P* value
Total duration of physical activity (h/day)				
Light (<3 METs)	0.034	0.608	0.022	0.746
Moderate (3–5.9 METs)	0.257	<0.001	0.225	0.001
Vigorous (≥6 METs)	0.354	0.481	0.330	<0.001
Duration of leisure-time physical activity (h/day)				
Light (<3 METs)	−0.018	0.790	0.008	0.910
Moderate (3–5.9 METs)	0.002	0.978	0.000	0.996
Vigorous (≥6 METs)	−0.048	0.474	−0.072	0.286
Duration of work (h/day)				
Sitting	−0.064	0.337	−0.133	0.047
Standing	0.165	0.013	0.256	<0.001
Walking	0.271	<0.001	0.239	<0.001
Heavy	0.376	<0.001	0.354	<0.001

MET: metabolic equivalent; TEE: total energy expenditure.

Partial correlation coefficients are adjusted for sex and age group.

## DISCUSSION

This study used the DLW method as a gold standard to examine the validity of a physical activity questionnaire designed for the Japanese population in a large number of subjects with widely varying physical activity levels. With the DLW method as the gold standard, JALSPAQ estimated TEE relatively well, but underestimation was more frequent at higher physical activity levels.

The body height and weight of the present subjects were similar to the standard values for the Japanese population.^18^ RMR was also similar to the standard RMR values for the Japanese population presented in Dietary Reference Intake.^18^ Thus, we conclude that the present subjects had the general physical characteristics of the Japanese general population. However, the physical activity level of the present subjects was higher than that noted in our previous studies: 42.9% of the present subjects were classified as active, using the definition in the Dietary Reference Intake.^18^ We recruited subjects at worksites requiring vigorous physical activity (ie, shipbuilding and hospitals). This may explain the higher physical activity level of the subjects.

Neilson et al reviewed a validation study of a physical activity questionnaire and suggested that, at the group level, the mean difference in TEE ranged from −800 to 1589 kcal/day (−3.35 to 6.65 MJ/day) and that the Spearman correlation coefficient for TEE ranged from 0.15 to 0.51.^2^ As compared with these results, JALSPAQ showed a smaller negative mean difference of −1.15 MJ/day and a higher correlation (Spearman correlation, 0.742; *P* < 0.001). A comparison of individual-level agreement indicates that the width of the 95% LOA in our study (7.68 MJ/day) was smaller than that in most other questionnaires described in the review of Neilson and colleagues (1133 to 17 948 kcal/day; 4.74 to 75.09 MJ/day).^2^ The relatively good agreement in this study partly resulted from the greater number of subjects (*n* = 226 in the present study vs *n* = 13 to *n* = 65 in previous studies) and the wide variation in TEE. Standard deviation was 2.77 MJ in the present study and 0.35 to 3.51 MJ in previous studies. A study by Racette showed the lowest 95% LOA (−2.42 to 0.16 MJ/day).^19^ However, that study was part of an investigation of a 17-week outpatient weight loss treatment, so the subjects were thought to be highly motivated and to have answered the questionnaire carefully. One reason why TEE is assumed to have greater accuracy than the existing questionnaire is that it is believed to have more detailed questions regarding occupational activity, housework, and leisure-time physical activity.

JALSPAQ tended to greatly underestimate TEE in more active subjects, possibly because the algorithm for the calculation of TEE for JALSPAQ only includes duration of time spent sitting, standing, and walking. These activities were scored on a scale from 1.5 to 4.0 METs. Even when there was a question regarding carrying heavy objects or engaging in activity of similar intensity, such activity was not used to calculate TEE. Thus, underestimation would be greater in subjects who expended considerable energy at work. In the present study, 16 subjects were engaged in shipbuilding, and the differences between TEE by DLW and JALSPAQ ranged from −10.98 to 0.34 MJ/day; TEE was overestimated by JALSPAQ in only 2 subjects.

Although TEE estimated by JALSPAQ showed a relatively good correlation with TEE by DLW, RMR accounted for a large part of TEE. To lessen the contribution of RMR, PAL was compared between the two methods. The results for PAL were poor, and individual differences were widely distributed. Therefore, JALSPAQ must either be improved or another new questionnaire should be developed to assess individual PAL.

We also attempted to identify a physical activity that characterized physical activity level. Our results showed that total time spent in moderate physical activity was significantly greater in the active group. In addition, moderate and vigorous physical activity had a weak but significant correlation with PAL. Thus, moderate physical activity is an important component of physical activity level, as Westerterp has suggested.^20^ However, the duration of moderate physical activity did not differ in the sedentary and moderate groups. Wareham et al used a very brief questionnaire that only included physical activity during work and recreational activities and found that physical activity ratio (daytime energy expenditure/resting metabolic rate), which was estimated using a heart rate monitor, did not differ between inactive and moderately inactive groups, even though VO_2max_ was different between these groups.^21^ Another method of classifying physical activity in sedentary subjects should thus be considered.

The present results also suggest that intensity and duration of physical activity during work (including occupational activity and housework) strongly affect PAL, whereas leisure-time physical activity does not. Both work and leisure-time physical activity play fundamental roles in total physical activity, which explains why previous brief physical activity questionnaires assessed only physical activity during work and leisure time.^21^^,^^22^ In the present study, because the mean duration of all leisure-time physical activity was 22 ± 21 minutes per day, the effect of leisure-time physical activity on TEE might be very small.

The most significant limitation of this study was that subjects were not selected randomly: they joined the study as volunteers. Hence, as compared with the general population, they might have remembered their physical activities better and completed the questionnaire more carefully. In addition, the variation in their physical activity level might differ from that of the general Japanese population. However, we were unable not determine the nature or extent of error that resulted from these subject characteristics. A second limitation is that the study periods for DLW and JALSPAQ were not identical. The DLW method determined the average TEE over 1 or 2 weeks. In contrast, JALSPAQ assessed typical physical activity over 1 month. This discrepancy could affect the validation of JALSPAQ. Finally, the relatively small proportion of sedentary subjects made it difficult to characterize the sedentary population. Although we tried to collect subjects with a broad range of physical activities, we could not collect comparable numbers of sedentary and active subjects.

In conclusion, PAL by JALSPAQ weakly correlated with PAL by DLW, although TEE by JALSPAQ was better correlated with TEE by DLW than with TEE assessed by the questionnaires used in previous studies. TEE underestimation was greater in active subjects than in sedentary and moderately active subjects. In addition, in this population, total moderate physical activity and physical activity during work were related to physical activity level, whereas leisure-time physical activity was not. To improve the physical activity questionnaire, an algorithm for heavy work should be added. In addition, to better differentiate sedentary subjects form moderate subjects, additional questionnaire items should be added or the algorithm should be reevaluated.
